# Host metabolome and faecal microbiome shows potential interactions impacted by age and weaning times in calves

**DOI:** 10.1186/s42523-023-00233-z

**Published:** 2023-02-14

**Authors:** Nida Amin, Sarah Schwarzkopf, Johanna Tröscher-Mußotter, Amélia Camarinha-Silva, Sven Dänicke, Korinna Huber, Jana Frahm, Jana Seifert

**Affiliations:** 1 HoLMiR - Hohenheim Center for Livestock Microbiome Research, University of Hohenheim, Stuttgart, Germany; 2 Institute of Animal Science, University of Hohenheim, Emil-Wolff-Str. 6-10, 70593 Stuttgart, Germany; 3 Institute of Animal Nutrition, Friedrich-Loeffler-Institut, Federal Research Institute for Animal Health, Brunswick, Germany

**Keywords:** Calves, Faeces, Age, Weaning time, Microbiome, Plasma metabolites

## Abstract

**Background:**

Calves undergo nutritional, metabolic, and behavioural changes from birth to the entire weaning period. An appropriate selection of weaning age is essential to reduce the negative effects caused by weaning-related dietary transitions. This study monitored the faecal microbiome and plasma metabolome of 59 female Holstein calves during different developmental stages and weaning times (early vs. late) and identified the potential associations of the measured parameters over an experimental period of 140 days.

**Results:**

A progressive development of the microbiome and metabolome was observed with significant differences according to the weaning groups (weaned at 7 or 17 weeks of age). Faecal samples of young calves were dominated by bifidobacterial and lactobacilli species, while their respective plasma samples showed high concentrations of amino acids (AAs) and biogenic amines (BAs). However, as the calves matured, the abundances of potential fiber-degrading bacteria and the plasma concentrations of sphingomyelins (SMs), few BAs and acylcarnitines (ACs) were increased. Early-weaning at 7 weeks significantly restructured the microbiome towards potential fiber-degrading bacteria and decreased plasma concentrations of most of the AAs and SMs, few BAs and ACs compared to the late-weaning event. Strong associations between faecal microbes, plasma metabolites and calf growth parameters were observed during days 42–98, where the abundances of *Bacteroides, Parabacteroides*, and *Blautia* were positively correlated with the plasma concentrations of AAs, BAs and SMs as well as the live weight gain or average daily gain in calves.

**Conclusion:**

The present study reported that weaning at 17 weeks of age was beneficial due to higher growth rate of late-weaned calves during days 42–98 and a quick adaptability of microbiota to weaning-related dietary changes during day 112, suggesting an age-dependent maturation of the gastrointestinal tract. However, the respective plasma samples of late-weaned calves contained several metabolites with differential concentrations to the early-weaned group, suggesting a less abrupt but more-persistent effect of dietary changes on host metabolome compared to the microbiome.

**Supplementary Information:**

The online version contains supplementary material available at 10.1186/s42523-023-00233-z.

## Background

The commercial calf rearing facilities are continuously challenged by cost reduction without affecting animal health and performance. Even a slight reduction in the weaning age can significantly reduce the feed cost. However, weaning age should be carefully considered as calves undergo extreme nutritional, metabolic, and behavioural changes from birth to the entire weaning period [1]. Feeding minimal plane of nutrition before weaning could result in long-term detrimental effects on calf’s growth and metabolic health [2]. The composition of the gut microbiome is unstable during the first three months of a calf’s life due to the change in physiological state, age, diet, weaning, and other environmental factors [3]. Besides other factors, pre-weaning calf diet contributes most strongly to the establishment of gut microbial communities and mucosal immune system [4]. The activity of gut microbes in turn benefit the host through digestion of complex dietary substrates, maturation of host immune system, intestinal epithelium development, maintenance of gut integrity and protection against pathogens [5–8]. The gut microorganisms produce a wide variety of metabolites either through direct fermentation of dietary substrates or through utilization of endogenous compounds produced by other gut microbes and the host [9]. These microbial metabolites are absorbed by the intestinal epithelium, enter the bloodstream to provide energy and nutrition to the host, regulate target organs and thus, alter the host’s metabolic state [10].

Most recent studies have highlighted the importance of integrating data from the microbiome and metabolome instead of solely microbial taxonomic profiling to better understand the host–microbe’s metabolic interactions and possible identifications of predictive biomarkers for diseases [11, 12]. With the advanced metabolomic analysis tools, it is now possible to detect several classes of metabolites such as amino acids (AAs), biogenic amines (BAs), acylcarnitines (ACs), and sphingomyelins (SMs) in a broad spectrum of matrixes such as blood or digestive material. These metabolites can provide a broader image of metabolic shifts and enable us to understand the underlying mechanisms caused by gut microbial dysbiosis [13]. Given the role of AAs in protein synthesis, energy generation and metabolic pathways regulation [14], plasma AAs quantification can provide an insight into the nutritional status, health and disease pathogenesis [15]. Similarly, high levels of BAs during rumen acidosis are regarded as a biomarker for bacterial dysbiosis [16], due to their important role in immunological, muscular, cardiovascular and neurological functions, as well as anti-inflammatory and anti-oxidative reactions [17]. Acylcarnitines were suggested as lipid mobilization biomarkers [18] and their high concentrations in plasma have been linked with both the healthy and diseased status of the host [19]. Sphingolipids are bioactive molecules, involved in several cellular and pathological processes including proliferation, cell division and differentiation, cell death, and pro-inflammatory responses [20]. Thus, it can be speculated that stress-related gut microbial dysbiosis can strongly impact the levels of metabolites [12]. To our knowledge, the association of gut microbiota with the plasma concentrations of AAs, BAs, ACs and SMs in pre- and post-weaned calves has not been examined so far. Although this evaluation should be done with care as a strong influence of host genetics on serum metabolites was described before [21], a more recent study found that 47% of the microbe-associated blood metabolites to be nonheritable [11]. This suggests the important role of gut microorganisms on the systemic metabolism, which is independent of the host’s genome. Here, we explored the changes in the calf’s faecal microbiome and plasma metabolome due to the developmental stage and the early and late-weaning event, inherently associated with qualitative and quantitative aspects of nutrient intake pattern.

## Results

### Age-dependent changes in the compositional profile of calves’ faecal microbiome

**Fig. 1:**
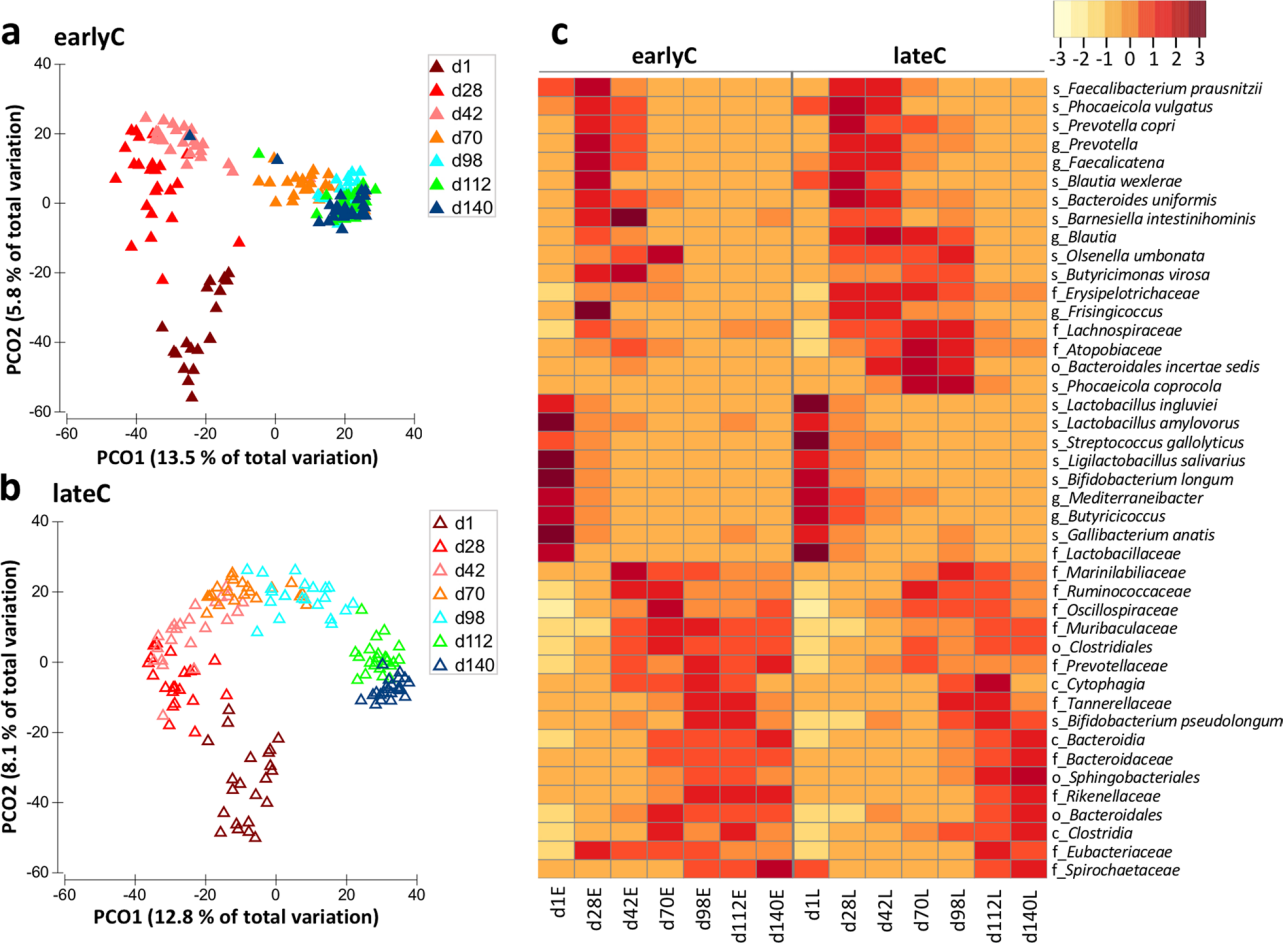
Age-dependent changes in the faecal bacterial communities of earlyC and lateC calves. **a**, **b** Bacterial compositional profiles of different age group earlyC (*n* = 176) and lateC (*n* = 154) faecal samples based on ASVs visualized using principal-coordinate analysis plots. Each triangle indicates one sample. **c** Heatmap based on bacterial taxa with significantly different relative abundance (*p* < 0.05; Kruskal–Wallis test). Each day represents an average value for animals: d1 (20 & 22), d28 (24 & 21), d42 (25 & 23), d70 (26 & 21), d98 (27 & 22), d112 (27 & 23), and d140 (27 & 22) animals for earlyC and lateC groups, respectively. EarlyC group was weaned during experimental days 28–42 and lateC during experimental days 98–112

The differences between the faecal bacterial community structure associated with age, weaning and parity of the mother were identified using Permutational Analysis of Variance (PERMANOVA) that showed a significant impact of age (*p* < 0.001), weaning time (*p* < 0.001), parity (*p* = 0.007) and the interaction between age and weaning time (*p* < 0.001) but parity was non-significant within the respective age and weaning groups. A clustering of bacterial communities based on amplicon sequence variants (ASVs) was observed by calves age in both weaning groups (Fig. 1A, B), which was further confirmed with the analysis of similarity test (ANOSIM) that showed significant differences between age groups (ANOSIM; *p* < 0.001; R = 0.65 and 0.75; earlyC and lateC, respectively). Both weaning groups showed a significant increase in faecal bacterial alpha-diversity with age (*p* < 0.001) as indicated by the lowest Shannon index values of 2.68 and 2.98 (d1) to the highest values of 4.94 and 4.95 (d140) in earlyC and lateC groups, respectively (Additional file 1: Fig. S1A). However, no significant impact of weaning time on diversity index was observed. With respect to the faecal bacterial taxonomic composition, a significant age-dependent decrease in the relative abundances of Firmicutes and Actinobacteria, while an increase in Bacteroidetes, Spirochaetes and Elusimicrobia was observed (Additional file 1: Fig. S1B, Additional file 2: Table S1). At species-level, the earliest time point (d1) had significantly higher abundances of *Bifidobacterium longum*, *Gallibacterium anatis*, *Lactobacillus amylovorus, Lactobacillus ingluviei, Ligilactobacillus salivarius, Streptococcus gallolyticus*, unclassified (uncl.) *Butyricicoccus*, uncl. *Lactobacillaceae*, and uncl. *Mediterraneibacter*, showing significant decrease in abundance with age (d1–d140) in both weaning groups. In addition, *Bacteroides uniformis, Barnesiella intestinihominis*, *Blautia wexlerae*, *Faecalibacterium prausnitzii*, *Phocaeicola vulgatus, Prevotella copri*, uncl. *Faecalicatena*, and uncl. *Prevotella* were significantly more abundant during days 28–42 and less abundant during later time points. On the contrary, *Bifidobacterium pseudolongum*, uncl. *Bacteroidia*, uncl. *Bacteroidales*, uncl. *Bacteroidaceae*, uncl. *Clostridia*, uncl. *Clostridiales*, uncl. *Eubacteriaceae*, uncl. *Muribaculaceae*, uncl. *Oscillospiraceae*, uncl. *Prevotellaceae*, uncl. *Ruminococcaceae*, uncl. *Rikenellaceae*, uncl. *Sphingobacteriales*, and uncl. *Tannerellaceae* were less abundant during early time points and showed a significant increase with age (Fig. 1C, Additional file 2: Table S1).

### Weaning-dependent modifications in the faecal bacterial composition and their predicted function in calves

**Fig. 2:**
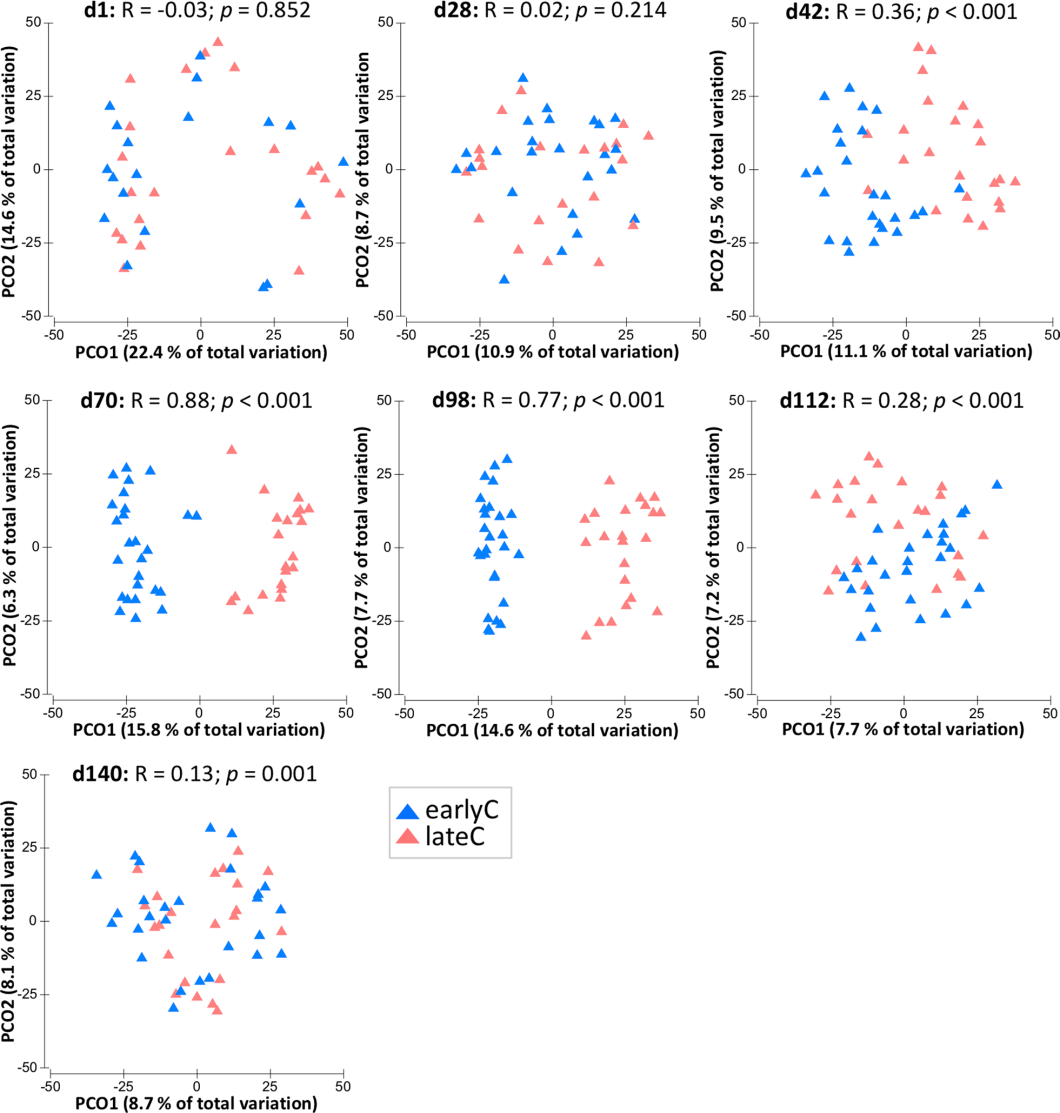
Principal-coordinate analysis plots showing changes in bacterial compositional profiles of faecal samples due to weaning event. Each triangle indicates one sample. The significant differences between same-age-old weaning groups, separated based on PCO analysis, were confirmed using analysis of similarities test (ANOSIM), with R- and *p* values indicated

**Fig. 3:**
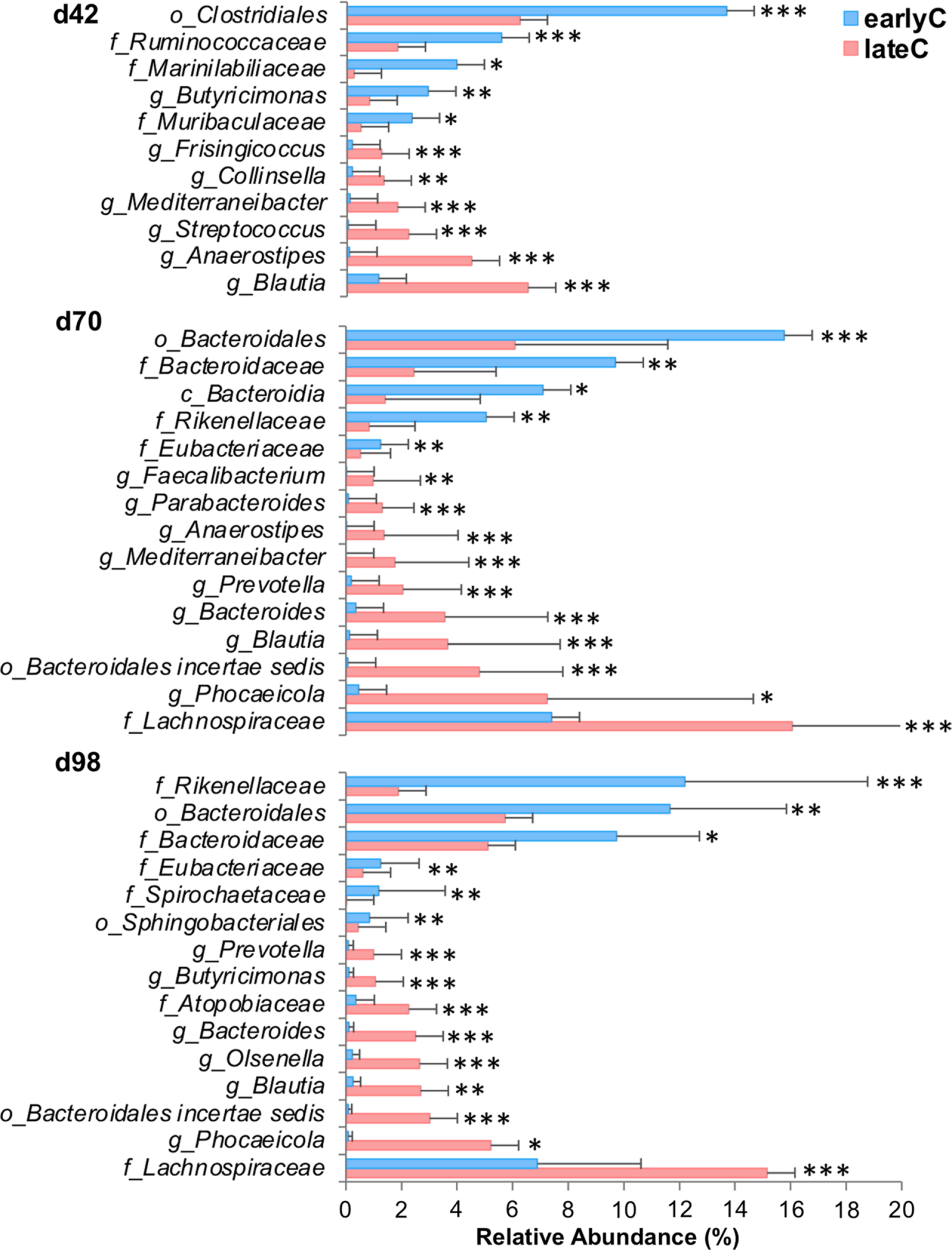
Changes in faecal bacterial communities of calves due to weaning event. Significantly different bacterial genera with relative abundance (≥ 1%) and *p*  ≤ 0.05 (Kruskal–Wallis test) among same-age old weaning groups are shown. Each bar represents an average value for animals: d42 (25 & 23), d70 (26 & 21), d98 (27 & 22) animals for earlyC and lateC groups, respectively

In addition to the age-related maturation, the time point of weaning also significantly influenced the faecal bacterial compositional profiles as indicated by the separate clustering of weaning groups during days 42–112. In contrast, no significant difference was detected before or after this period (Fig. 2). Both weaning groups had distinct bacterial taxonomic compositions during days 42–98 (Fig. 3, Additional file 2: Table S1). Early-weaning at seven weeks triggered an increase in the relative abundance of Bacteroidetes and a decrease of Firmicutes (Additional file 1: Fig. S1B) during days 42–98 (*p* < 0.05). At genus-level, earlyC calves had significantly higher abundances of *Butyricimonas* and certain unclassified members of Bacteroidetes, Firmicutes, as well as Spirochaetes (Fig. 3, Additional file 2: Table S1). Early-weaning also significantly decreased the abundances of potential lactose- and starch-degraders as well as potential butyrate-producing bacteria including *Faecalibacterium*, *Blautia, Prevotella, Bacteroides, Parabacteroides, Butyricimonas, Olsenella*, *Anaerostipes, Streptococcus, Frisingicoccus, Phocaeicola, Mediterraneibacter*, uncl. *Atopobiaceae*, uncl. *Bacteroidales incertae sedis*, and uncl. *Lachnospiraceae.* In addition, the abundance of potential pathogenic bacteria, such as *Collinsella*, was reduced due to the weaning event in the earlyC group.

CowPI-based predictive analysis showed a significant enrichment of function with particular involvement in the metabolism of amino acid, carbohydrate, energy and nucleotide, and glycan biosynthesis in the earlyC group (days 42–98) (Additional file 1: Fig. S2). In contrast, a significant reduction in some of the general metabolic functions with essential role in microbial survival such as protein kinases, ABC transporters, two-component system, transcription factors, and other ion-coupled transporters were also predicted in the earlyC group corresponding to the weaning event.

### Plasma metabolome and the impact of calves’ age

**Fig. 4:**
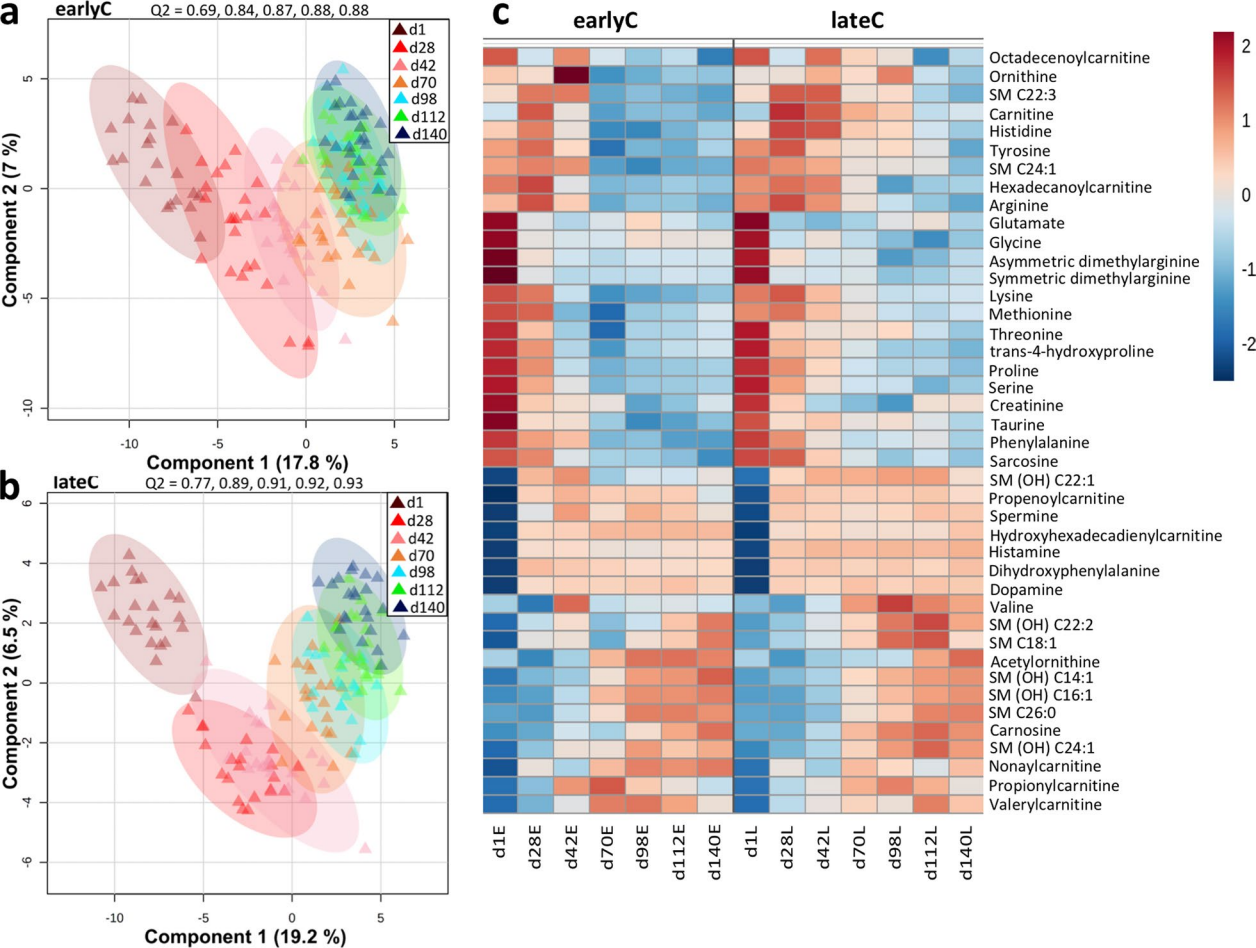
Age-dependent changes in plasma metabolites concentrations of earlyC and lateC calves. **a**, **b** Metabolic profiles of different age group earlyC (*n* = 174) and lateC (*n* = 153) plasma samples visualized using PLS-DA score plots. Each shape indicates one sample coloured according to the age group with ellipse indicating the 95% confidence region. **c** Heatmap of the significantly altered metabolites due to calves age (VIP > 1, FDR < 0.001, ANOVA). Each day represents an average concentration of metabolites for animals: d1 (20 & 22), d28 (24 & 21), d42 (24 & 23), d70 (25 & 21), d98 (27 & 21), d112 (27 & 23), and d140 (27 & 22) animals for earlyC and lateC groups, respectively

The differences between the plasma metabolic profiles of calves from different age groups were shown by a supervised partial least square discriminant analysis (PLS-DA), that resulted in clear age-dependent clustering for both weaning groups (Fig. 4A, B). Metabolites showing significant difference due to the age of the calves were selected based on the variable importance in the projection (VIP) threshold > 1 and a false discovery rate (FDR) < 0.001 (ANOVA) (Fig. 4C, Additional file 2: Table S2). The plasma concentrations of most of the metabolites including AAs, BAs, ACs, and SMs were affected by both calves age and the time of weaning (Fig. 4C). In both weaning groups, a significant age-dependent decrease in the concentrations of AAs (arginine, lysine, methionine, phenylalanine, threonine, proline, serine, tyrosine, glutamate, glycine, and histidine), BAs (taurine, trans-4-hydroxyproline, creatinine, sarcosine, asymmetric dimethylarginine, and symmetric dimethylarginine), AC (carnitine) and SM (SM C24:1) was observed. However, as the calves aged and became more mature (days 70–140), the plasma concentrations of BAs (carnosine, acetylornithine, dopamine, spermine, histamine, and dihydroxyphenylalanine), ACs (hydroxyhexadecadienylcarnitine, and valerylcarnitine), and most of the sphingomyelins (SM (OH) C14:1, SM (OH) C16:1, SM (OH) C22:1, SM (OH) C22:2, SM (OH) C24:1, SM C18:1, SM C26:0) were increased (Fig. 4C).

### Weaning-dependent modifications in the plasma metabolome of calves

**Fig. 5:**
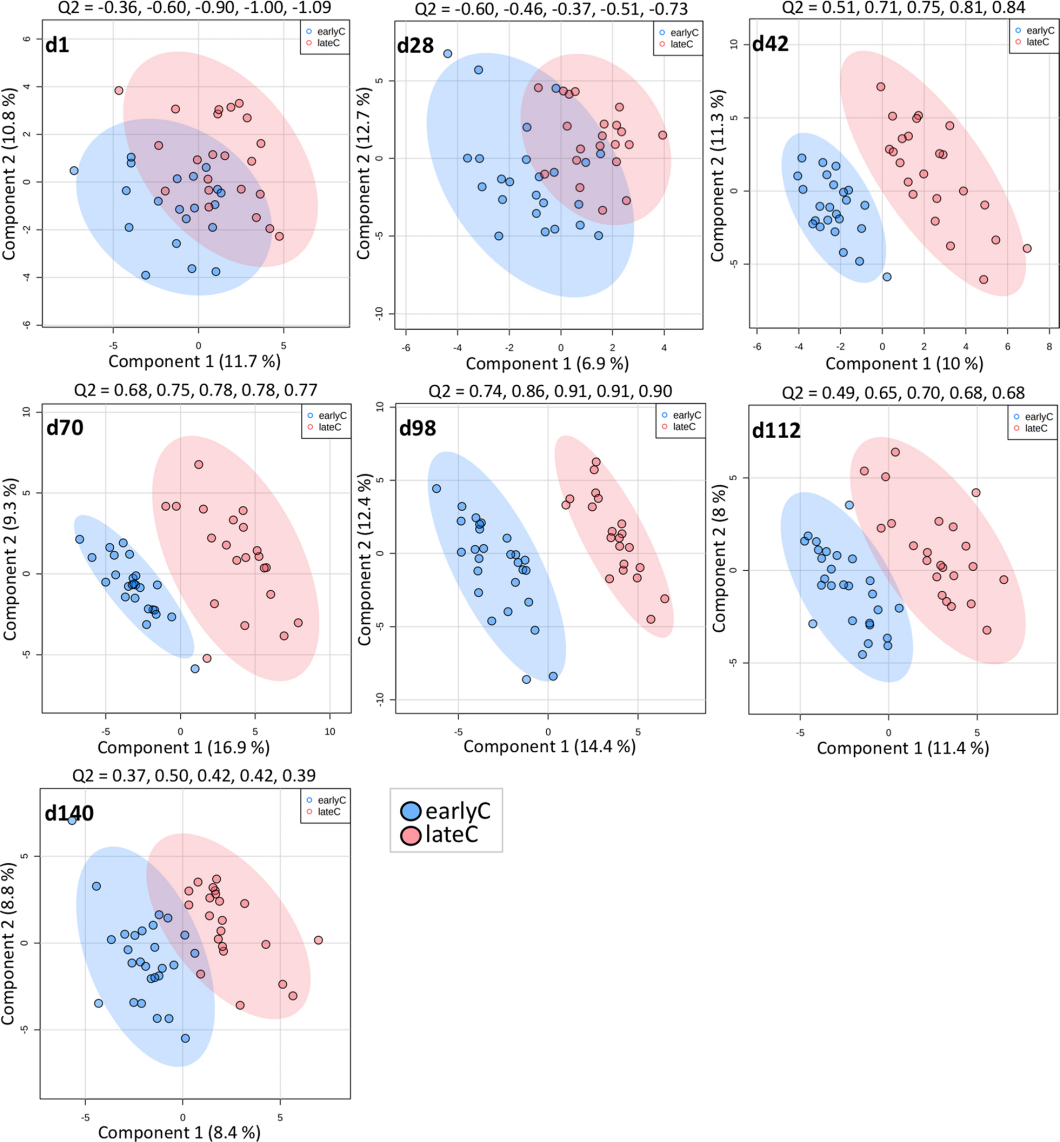
Partial least squares-discriminate analysis for identification of metabolic differences among weaning groups. Each circle indicates one sample and ellipse indicating the 95% confidence region. The quality of the models was assessed using Q2 as performance measure and tenfold cross-validation method. The Q2 values for the first 5 components are shown

**Fig. 6:**
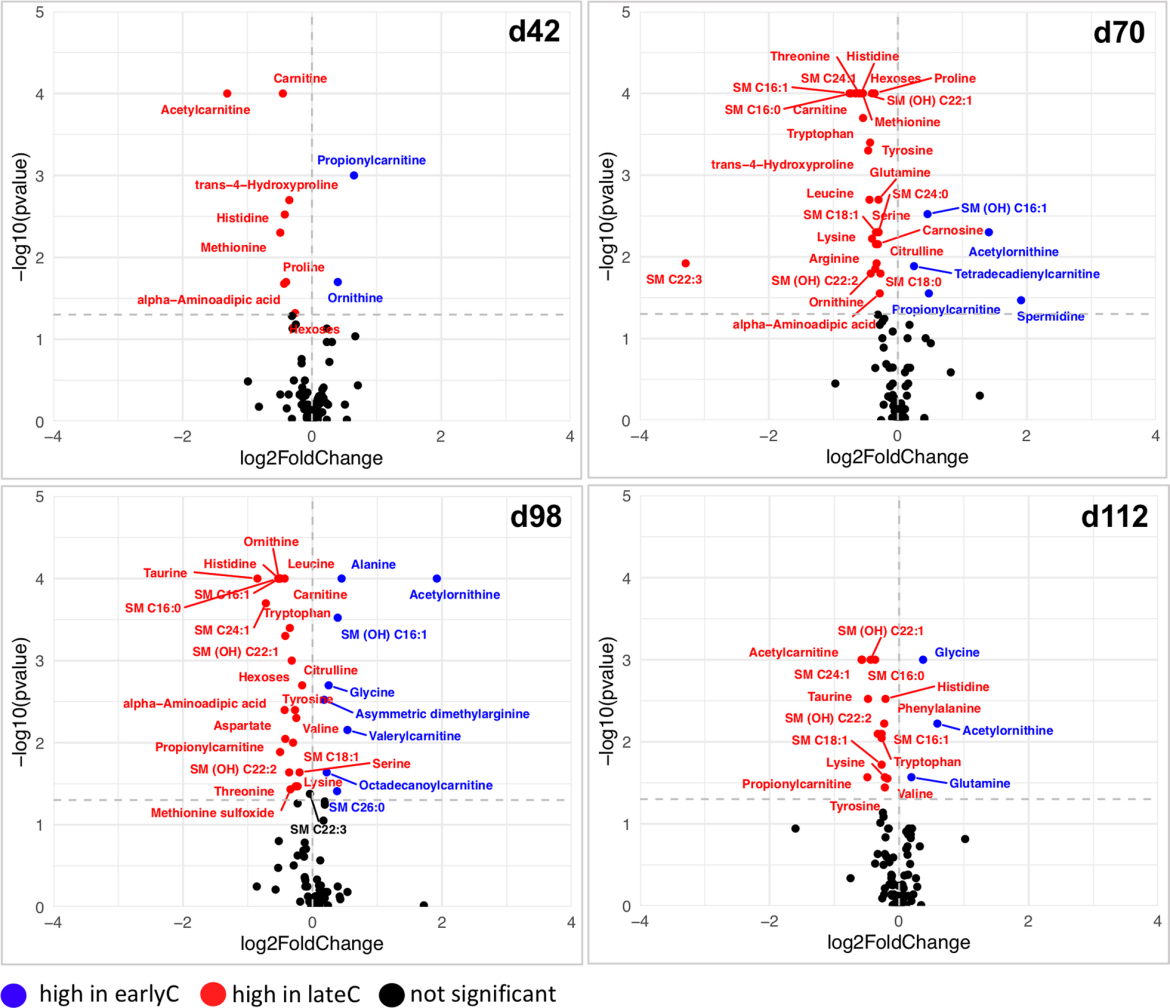
Volcano plots of the weaning-dependent changes in the plasma metabolic profiles of weaning groups. The identification of significantly altered metabolites due to weaning event within each age group was based on a VIP > 1, FDR < 0.05 (*t*-test) and log2 FC > 0.1 or < − 0.1. Each circle indicates one metabolite

Similar to the weaning-related shifts in the faecal microbial profiles, the supervised PLS-DA showed clear separation among metabolic profiles of earlyC and lateC calves during days 42–112 (Fig. 5). The identification of metabolites altered due to the weaning event within each age group was based on a VIP > 1, FDR < 0.05 (*t*-test) and log2 FC > 0.1 or < − 0.1. Mother’s parity showed no significant influence on DMs within each weaning group (earlyC PC vs. earlyC MC and lateC PC vs. lateC MC). A total of 10, 32, 32, and 18 significantly differential metabolites (DMs) were identified between earlyC and lateC groups at days 42, 70, 98, and 112, respectively. During days 42–112, the relative concentrations of 2, 5, 8 and 3 metabolites were significantly higher in the plasma of earlyC calves, and the relative concentrations of 8, 27, 24, and 15 metabolites were significantly higher in the plasma of lateC calves (Fig. 6). In general, earlyC calves had significantly lower concentrations of most of the essential amino acids (EAAs; arginine, histidine, leucine, lysine, methionine, phenylalanine, valine, threonine, tryptophan), and non-essential amino acids (NEAAs; aspartate, glutamine, proline, serine, tyrosine, citrulline, and ornithine), BAs (taurine, trans-4-hydroxyproline alpha-aminoadipic acid, carnosine, and methionine sulfoxide), ACs (carnitine, acetylcarnitine, and propionylcarnitine), and SMs (SM (OH) C22:1, SM (OH) C22:2, SM C16:0, SM C16:1, SM C18:0, SM C18:1, SM C22:3, SM C24:0, SM C24:1) as compared to the same-day-old lateC group (days 42–112; Fig. 6). The ratio between kynurenine/tryptophan was lower at day 70 and 98 (Additional file 1: Fig. S3) in the lateC group. Similar to the microbiome dataset, no significant differences between metabolic profiles of weaning groups were observed during days 1–28, but the plasma samples of 112 days old earlyC and lateC calves showed a large number of DMs. A metabolic pathway analysis (MetPA) was done using DMs identified between the weaning groups. The enrichment of 5 (d42), 12 (d70), 13 (d98), and 9 (d112) pathways mainly related to AAs metabolism was shown to be significantly different between the weaning groups (Additional file 1: Fig. S4, pathway impact ≥ 0.1, FDR < 0.01).

### Associations between differential faecal microbial genera and plasma metabolites of weaning groups

**Fig. 7:**
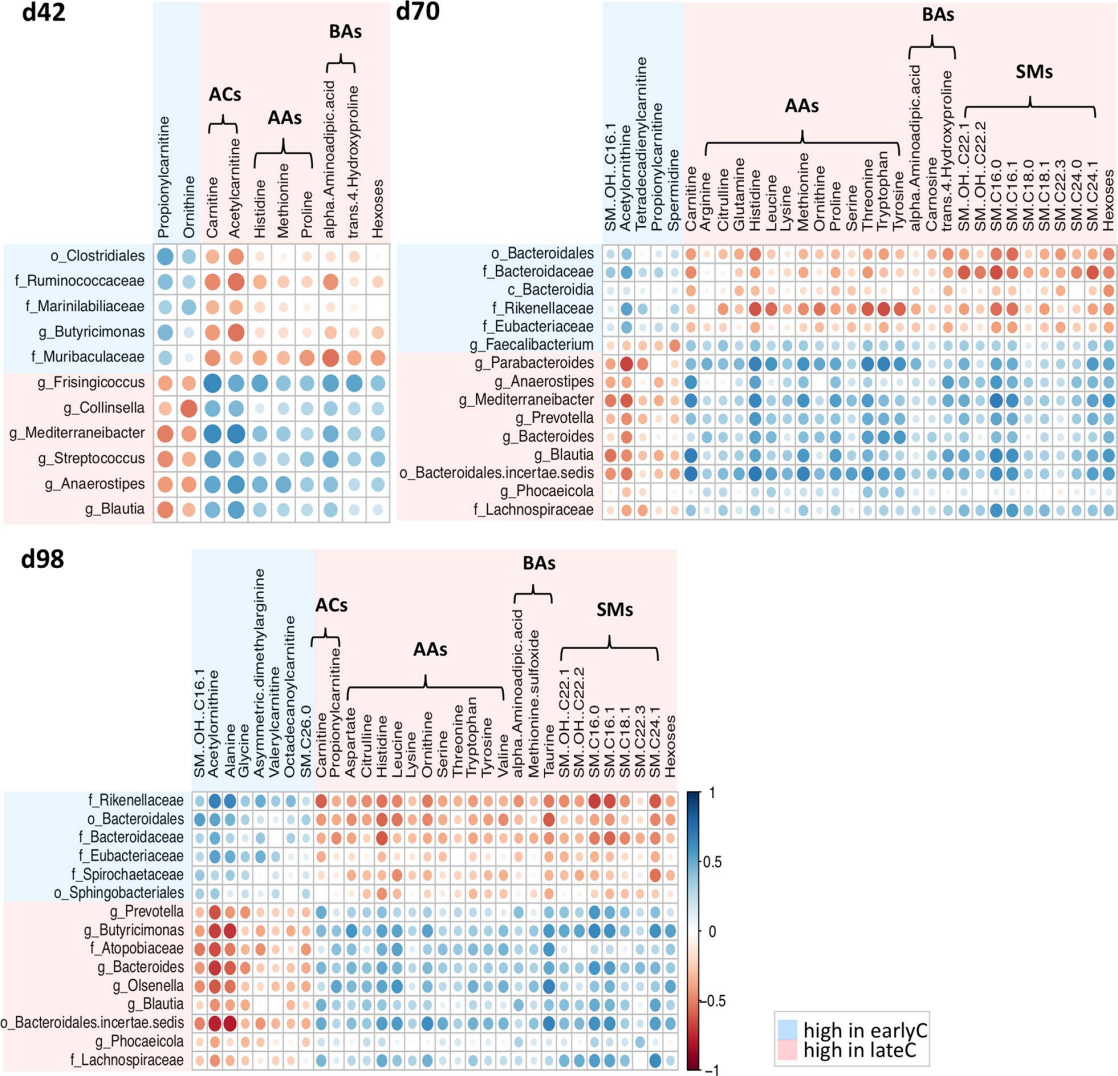
Heatmaps showing the Spearman’s rank correlations between differentially abundant faecal microbial genera and plasma metabolites of weaning groups. Colours indicates the correlation between microbiome and metabolome (blue: significant positive, red: significant negative, and white: non-significant). Only Spearman correlation coefficients with *p* < 0.05 are shown. Abbreviations (ACs, AAs, BAs and SMs) indicates following metabolites classes: acylcarnitines, amino acids, biogenic amines, and sphingomyelins respectively

To identify the weaning-dependent shifts in the potential host-microbe metabolic interactions, Spearman’s rank correlations were calculated between the differentially abundant faecal microbial genera and plasma metabolites of weaning groups, separately for each time point (Fig. 7). The potential lactose- and starch-degrading bacterial genera that were reduced by the early-weaning events during days 42–98 were strongly positively correlated (R > 0.50, *p* < 0.05) with the plasma concentrations of AAs, BAs and SMs. Aspartate was positively correlated with *Butyricimonas*, histidine with *Frisingicoccus, Blautia, Bacteroides, Prevotella, Mediterraneibacter, Anaerostipes, Parabacteroides, Butyricimonas* and *Olsenella*, methionine and proline with *Blautia, Mediterraneibacter* and *Parabacteroides*, leucine and ornithine with *Parabacteroides* and *Butyricimonas*, threonine, tryptophan and tyrosine with *Bacteroides* and *Parabacteroides*, leucine with *Olsenella*, and threonine *with Mediterraneibacter.* Similar positive correlations were observed between the plasma concentrations of BAs such as alpha-aminoadipic acid with *Frisingicoccus*, taurine with *Bacteroides, Butyricimonas* and *Olsenella*, trans-4-hydroxyproline with *Frisingicoccus*, *Blautia, Mediterraneibacter* and *Anaerostipes.* The plasma SMs concentrations were positively correlated with bacterial abundances; SM (OH) C22:1 with *Blautia, Mediterraneibacter* and *Butyricimonas*, SM (OH) C22:2 with *Butyricimonas*, SM C24:1 with *Mediterraneibacter, Parabacteroides, Blautia* and *Butyricimonas*, SM C16:0 with *Blautia, Prevotella, Mediterraneibacter, Anaerostipes, Parabacteroides, Bacteroides* and *Butyricimonas*, and SM C16:1 with *Blautia, Mediterraneibacter, Bacteroides* and *Butyricimonas* (Fig. 7).

In addition, the genera that were significantly higher in abundance in the earlyC group during days 42–98 were also strongly positively correlated to the following plasma metabolites: uncl. *Rikenellaceae* with alanine, uncl. *Clostridiales* with propionylcarnitine, uncl. *Bacteroidales, Bacteroidaceae, Rikenellaceae* and *Eubacteriaceae* with acetylornithine, and uncl. *Bacteroidales* with SM (OH) C16:1 (R > 0.50, *p* < 0.05). Furthermore, few strong negative correlations also existed between uncl. *Bacteroidales* with histidine, leucine, taurine, SM C16:0, and SM C16:1, uncl. *Bacteroidaceae* with SMs (SM (OH) C22:1, SM (OH) C22:2, SM C16:0, SM C16:1, and SM C24:1) as well as with AA (histidine), uncl. *Muribaculaceae* with alpha-aminoadipic acid, uncl. *Rikenellaceae* with carnitine, AAs (histidine, leucine, ornithine, threonine, tryptophan, tyrosine), taurine, and SMs (SM C16:0, SM C16:1, and SM C24:1), uncl. *Ruminococcaceae* and *Butyricimonas* with acetylcarnitine, uncl. *Spirochaetaceae* with SM C24:1 (R < − 0.50, *p* < 0.05).

### Associations between morphometric variables of calves, differential faecal microbial genera and plasma metabolites of weaning groups

Live weight (LW), live weight gain (LWG) or average daily gain (ADG), and morphometric variables such as withers height, hip height, back length, heart girth and body length increased with age (*p* < 0.001) and were higher in the lateC group [22]. The data were checked for strong positive (R > 0.50) or strong negative correlations (R < − 0.50; *p* < 0.05, Additional file 2: Table S3) with microbiome and metabolome data. LWG or ADG was significantly higher for lateC group from days 42–98, showing strong positive correlations with the abundances of *Mediterraneibacter, Parabacteroides, Prevotella, Blautia*, uncl. *Bacteroidales incertae sedis*, uncl. *Lachnospiraceae* (d70), and *Olsenella* (d98), as well as the plasma concentrations of threonine, tryptophan, tyrosine, histidine, methionine, proline, carnitine, hexoses, SM C16:0, and SM C16:1 (d70), while strong negative correlation with uncl. *Rikenellaceae* abundance and acetylornithine concentration (d70) were observed. LateC group had significantly higher LW from days 70–140, which was strongly positively correlated with uncl. *Bacteroidales incertae sedis* (d70 and d98), uncl. *Atopobiaceae* (d98), and plasma concentrations of methionine, serine, trans-4-hydroxyproline, and carnitine (d70), tryptophan, tyrosine, valine, leucine, ornithine, taurine, hexoses, SM C24:1, SM C16:0, SM C16:1 (d98), and threonine (d70 and d98), and negatively correlated with uncl. *Rikenellaceae* (d98), spermidine (d70) and acetylornithine (d98). Hip height was significantly different between the weaning groups only during days 70 and 140, and positively correlated with the abundances of *Blautia, Mediterraneibacter, Prevotella* and uncl. *Bacteroidales incertae sedis*, and plasma concentrations of lysine, threonine, histidine, methionine, serine and carnitine, while negatively correlated with spermidine and SM (OH) C16:1 (d70). Heart girth was greater for lateC group from days 98 onwards and had a strong positive association with the abundance of unclassified *Bacteroidales incertae sedis*, plasma concentration of tryptophan, valine, leucine, ornithine, SM C24:1, SM C16:0 and SM C16:1, and strong negative association with uncl. *Rikenellaceae* abundance and acetylornithine (d98).

## Discussion

This study examined the age- and weaning-dependent changes in the calves’ faecal microbiome, plasma metabolome and explained the potential host-microbe associations. We showed an age-dependent increase in the faecal bacterial alpha-diversity as reported in other studies [23, 24], which might have assisted GIT development and liquid to solid diet transition post-weaning [25]. At species-level, the faecal bacterial community of young calves was dominated by potential lactose- and starch-degrading bacteria, which was replaced by potential fiber-degrading bacteria with age. A similar age-related decrease in the abundances of *Bifidobacterium*, *Lactobacillus*, and *Faecalibacterium* [25, 26], and an increase in fiber-degrading *Ruminococcus* was recently reported [25]. Bifidobacteria can utilize carbohydrates freely available in the pre-weaned calf GIT [27] and are usually isolated from faecal samples of new born calves, and young ruminants [27–29]. Similarly, *F. prausnitzii* was found in faecal samples of 3–4-week-old calves, showing an age-dependent decrease in abundance as observed in our study [30]. The high abundance of *F. prausnitzii* has also been linked with increased weight gain and lower incidence of diarrhoea in dairy heifers and Holstein calves during the pre-weaning period [23, 31]. We also reported an age-dependent decrease in certain potential pathogenic bacteria such as *Streptococcus gallolyticus*, found in newborn calves with purulent lesions and meningitis [32], and *Gallibacterium anatis*, isolated from cattle with respiratory diseases [33], indicating an age-dependent maturation of the immune system in calves.

In addition to the age, the time at which animals were weaned (7 or 17 weeks of age) had an important role in shaping their gut microbial communities. The major differences between the bacterial composition of weaning groups were observed during days 42–98. The earlyC group was characterized by a significantly higher abundance of phylum Bacteroidetes and potential fiber-degrading bacteria. In contrast, the lateC group was dominated by Firmicutes and potential lactose- and starch-degraders. The differential bacterial composition of weaning groups during days 42–98 was due to their different feed intake pattern as described previously [22]. During day 42, lateC group had higher milk replacer (MR) intake, while earlyC had higher roughage and concentrate (C) intake. However, during days 70–98, earlyC group was characterized by a total mixed ration (TMR) feeding pattern, while, the lateC group still consumed substantial amounts of MR and C. Castro and colleagues suggested that the increase in MR intake may result in higher lactose flux in the hindgut, serving as a prebiotic and a growth substrate for certain beneficial microorganisms [34]. In accordance with this study, the faecal microbiota of lateC calves (days 42–98) had high dominance of *Bacteroides, Prevotella, Faecalibacterium*, *Butyricimonas, Blautia*, and *Olsenella*. Few other studies have reported an increased dominance of *Bacteroides, Prevotella, Faecalibacterium* and *Blautia* in MR-fed pre-weaned calves’ faeces [35]. Likewise, a positive association between MR intake and faecal *Blautia* abundance in pre-weaned calves [36] and a negative association between dietary forage inclusion and faecal *Bacteroides, Olsenella* abundances have been reported [37, 38]. The high abundance of *Bacteroides*, *Faecalibacterium*, and *Butyricimonas* has also been linked with lower disease susceptibility in calves [39, 40]. Thus, it can be speculated that the decrease in the abundances of major lactic-acid producing bacteria with age and after day 42 in the earlyC group was due to their increased fiber ingestion and the decreased milk consumption, resulting in limited nutrient availability for the growth of potential lactose- and starch-degrading microorganisms. In addition to the beneficial microorganism, we also observed a significantly higher abundance of pathogenic bacterial genus *Collinsella* in 42-day-old lateC calves’ faeces. This bacterial genus reduces the expression of tight junctions and increases intestinal permeability, resulting in gut leakage and pro-inflammatory dysbiosis [41, 42]. Their abundance was linked with host dietary intake, such as higher abundance in MR-fed calves’ faeces [43] and lower abundance with fiber-rich diet [44]. Thus, the low abundance of *Collinsella* in 42-day-old earlyC group in our study was probably due to the introduction of roughages in their post-weaning diet. Moreover, no significant differences in the bacterial composition of the weaning groups were observed during later time points (days 112–140) indicating a rapid adaptation of the lateC microbiome to the weaning-related dietary changes without causing dysbiosis.

Besides the differences described above, the plasma metabolic profiles of calves also showed age- and weaning-dependent modifications. The plasma samples of young calves (days 1–28) had high concentrations of most of the AAs, but their concentrations declined with age and after weaning event in the earlyC group. The plasma AAs concentrations are dependent on many factors such as synthesis and breakdown of proteins, and it is known that highly digestible milk protein levels lead to an improved AAs absorption which results in higher blood levels [45]. A high plasma concentration of EAAs and NEAAs was observed after MR-feeding in Holstein bull calves [46]. Similarly, feeding a high amount of milk during the pre-weaning period increased the levels of plasma arginine and lysine in Holstein heifer calves [47], suggesting that the liquid diet could provide specific metabolites that can be transported into the bloodstream through GIT [48]. In ruminants, depending on the stage of development, digestion and fermentation takes place in different sections of the GIT. Neonatal ruminants mainly rely on their hindgut for digestion of feed and metabolites synthesis [49], this restricts the absorption of certain metabolites as the absorption capacity in the colon is limited. With the development of the rumen, the major microbial activity is located in the forestomach and the microbial metabolites are absorbed through the epithelium of the rumen or the lower GIT and supply energy to the host [50, 51]. Therefore, a lower level of plasma metabolites at the early life of a ruminant is true to the fact of the limited absorption capacities in the hindgut and have to be considered for the interpretation.

Not only the plasma AAs concentrations were affected, but we also observed distinct profiles of BAs at different developmental stages. The early-weaning event lowered the concentrations of certain BAs (taurine, trans-4-hydroxyproline, alpha-aminoadipic acid, carnosine, and methionine sulfoxide) as well as plasma ACs (carnitine, acetylcarnitine, and propionylcarnitine) compared to the late-weaning event. The difference in plasma BAs and ACs concentrations of weaning groups was probably due to their different dietary composition as the carbohydrates rich diet may result in higher levels of BAs [52]. A high concentration of serum taurine was observed in high-grain fed dairy cows [53]. A decreased level of plasma acylcarnitines was observed after feeding calves with a limited amount of MR in another study [2]. Similar to the AAs, BAs and ACs, the plasma concentrations of most of the SMs were also lower in the earlyC compared to the lateC group. The functional aspects of the changed sphingomyelin profile in calves are still unclear, however, lower concentrations of blood SMs (SM OH C14:1 and SM OH C16:1) were linked with metabolic stress in periparturient cows [54]. It may be assumed that the lower level of plasma SMs in earlyC calves was probably due to the stressful weaning event as the animals were not fully matured and sudden dietary changes might have resulted in quick transitioning from a non-ruminant to a pre-ruminant. Contrary to the microbiome dataset that had no significant differences between samples of 112-day-old early- and late-weaned calves, the plasma revealed several metabolites with differential concentrations, suggesting that the weaning related-dietary changes had less abrupt but more-persistent impact on host metabolism compared to the microbiome.

The associations between the faecal microbial genera, plasma metabolites and calf growth parameters were assessed during the weaning event to track the weaning-dependent modifications in the potential host-microbe metabolic interactions. LWG or ADG was higher in the lateC group during days 42–98 and correlated with the faecal abundances of *Parabacteroides, Blautia*, *Mediterraneibacter, Olsenella, Prevotella*, and the plasma concentrations of histidine, threonine, tryptophan, tyrosine, methionine, proline, carnitine, hexoses, SM C16:0, and SM C16:1. High abundances of *Blautia* and *P. copri* were observed in steers with high ADG [55] and a positive correlation between *Blautia*, *Prevotella* abundances and ADG was recently reported [36, 56], indicating the importance of these bacterial group for ruminants. The early-weaning event not only decreased the LWG or ADG, but the plasma concentrations of most of the AAs, BAs and SMs as well as the abundances of several potential lactose- and starch-degrading bacteria were reduced. Plasma AAs are essential for health and an alteration in their concentrations may result in immune responses and inflammation. Proline possesses antioxidant properties and protects against reactive oxygen species [57]. Leucine involvement in tissues and cells protein synthesis was previously reported in pigs and mice [58, 59]. Tryptophan and its degradation product kynurenine are used as indicators for low-grade chronic inflammation in humans [60]. Here, lateC animals had lower ratio at d70 and d98, which is indicates a possible increased inflammatory status of the earlyC animals during this time period and matches to previous findings reporting a lower kynurenine/tryptophan in healthy dairy cows [19]. The lower plasma levels of arginine, glutamine, methionine, histidine have been linked with the increased incidence of diarrhoea in calves [61]. Our study reported that the weaning event affected the predicted AAs metabolic pathways, specifically during days 42–98. At the same time, higher plasma concentrations of histidine, threonine, tryptophan, and tyrosine were measured in the lateC group. These AAs were positively correlated with the abundances of *Bacteroides* and *Parabacteroides.* Similar trends were observed with methionine, proline, and histidine concentrations that were positively correlated with *Blautia* abundance, while the concentrations of leucine, ornithine, methionine, and proline, were positively associated with the *Parabacteroides*. *Bacteroides* members are essential for AAs metabolism in the large intestine [62]. Similarly, *Parabacteroides*, which was assigned to the *Bacteroides* genus prior to reclassification in 2006 [63], also produces a wide range of AAs such as alanine, glutamate, histidine, isoleucine, lysine, methionine, phenylalanine, proline, and valine [64]. A recent study also reported the significant correlation of *Bacteroides* and *Blautia* abundances with the faecal metabolites involved in AAs metabolism (proline, and leucine) [65]. *Butyricimonas* abundance was relatively higher in the lateC group at day 98 and it was positively correlated with plasma aspartate concentration. Similar positive association between *Butyricimonas* and N-acetylaspartate was reported in young pigs [66]. The lower plasma AA levels and their associations with diet-related diminished abundance of AAs producing bacteria in the earlyC group is understandable. However, the identification of the causal relationships of the observed correlations are challenging as the plasma AA concentrations are not only determined by diet but also strongly by liver and muscle metabolisms which are yet to be explored.

In addition to the AAs, the plasma concentration of taurine and faecal *Bacteroides* abundance was significantly higher in the lateC group during day 98 and were positively correlated with each other. Similar to our study, a high dominance of *Bacteroides* in MR-fed pre-weaned calves’ faeces [35] and its negative association with dietary forage inclusion have previously been reported [38]. Taurine can be derived directly from the diet, absorbed through the epithelial cells and transported to the blood [17]. A significant increase in serum taurine concentration was reported with high-grain feeding in dairy cows [53]. However, endogenous synthesis of taurine from methionine and cysteine majorly takes place in liver and tissues [67]. Taurine is released into the gut as conjugated bile salts [68], where it is deconjugated by bacterial bile salt hydrolases (BSH) [69], expressed by several member of *Bacteroides* (*B. vulgatus* and *B. uniformis*) [70]. This process increases the concentrations of bile salts and taurine in the lower digestive tract [71], which can further be absorbed from the distal ileum and transported to the blood as reported in recent human study [17]. Taurine plays an essential role in regulation of gut micro-ecology through inhibition of potential pathogenic bacteria, reduction of lipopolysaccharides concentrations and acceleration of SCFA synthesis [72]. The association of plasma taurine concentration with liver functionality has previously been reported in cows [73]. This confirms our previous findings reporting lower liver cholesterol production to compensate weaning-related dietary lack in earlyC group as compared to the lateC group [22]. Thus, it can be speculated that the weaning-dependent addition of dietary roughages might have resulted in lower availability of dietary taurine, reduced abundances of bile salt hydrolysing bacterial genera and the resultant lower absorption of taurine from the gut due to insufficient BSH activity in the 98-day-old earlyC group.

In addition, the plasma concentrations of several SMs (SM (OH) C22:1, SM (OH) C22:2, SM C24:1, SM C16:0, SM C16:1) were significantly higher in the lateC group and positively associated with the abundances of *Bacteroides*, *Parabacteroides, Prevotella, Anaerostipes, Blautia*, *Butyricimonas*, and *Mediterraneibacter* during days 70–98. *Bacteroides, Parabacteroides*, and *Prevotella* are sphingolipids (SLs)-producing bacterial genera [74]. *Bacteroides* are among the few bacteria that can synthesize SLs and utilize them to survive in the stressful intestinal environment [75]. The *Bacteroides* members produce SLs-rich outer membrane vesicles (OMVs) [76], which are described to penetrate the intestinal mucosa and exert immune-related effects on the host [77]. Some recent studies reported the possible processing of *Bacteroides*-SLs via mammalian SL pathways [78] and the utilization of bacteria-derived SLs during food deprivation periods [74]. Thus, the higher abundance of SLs-producing bacteria in the lateC group might be one of the contributing factors towards their higher plasma SMs concentrations. Hence, a change in the composition of faecal microbiome and plasma metabolic profiles over the course of development, the higher abundances of several beneficial bacterial genera in lateC group and their positive association with AAs, BAs and SMs concentrations suggesting that the gut microbial colonization might play a certain role in this phenomenon.

## Conclusion

Our study showed that the progressive development of faecal microbiome and plasma metabolome in calves depends on their developmental stage and the time of weaning. A high dominance of potential lactose- and starch-degrading bacteria and a high concentration of the plasma AAs and BAs were observed in young calves, but as the calves aged, the abundances of unclassified members of potential fiber-degrading bacteria and the plasma concentrations of SMs and few BAs and ACs were increased. Higher consumption of roughages at day 42 in the earlyC group declines the abundances of potential lactose- and starch-degraders, and the plasma concentrations of most of the AAs and SMs, few BAs and ACs. This weaning-dependent modification in the microbiome composition and plasma metabolic profiles of calves were significantly correlated. On the contrary, the faecal microbial communities of lateC group showed quick adaptability to the weaning-dependent dietary changes, indicating an established microbial consortium compared to the earlyC group. Nevertheless, the plasma samples of lateC group at day 112 showed several metabolites with differential concentrations to the earlyC group, suggesting that the weaning-dependent dietary changes had a less abrupt but more-persistent impact on host metabolome compared to the microbiome. Altogether, the integration of faecal microbiome and plasma metabolome provided us initial insight into the host–microbe’s interactions in calves during weaning. However, the plasma metabolic profiles are not only dependent on diet and microbiome, but are also linked to liver and muscle metabolism, as well as the host genetics. Therefore, further studies are needed, where the associations between gut microbiome, gut metabolome, blood metabolome, liver and muscle metabolism must be explored to better understand the role of the microbiome in host metabolism and possible identifications of predictive biomarkers for diseases.

## Methods

### Animals and experimental procedures

The experiment was performed using 59 female German Holstein calves, raised under controlled environmental conditions from birth until 149 ± 2 days of life. The experimental design was the same as described previously [22]. Briefly, the experimental period started when calves were 8 ± 1.9 days old. Calves were randomly allocated into two weaning groups, weaned at 7 weeks (experimental days 28–42, earlyC) and 17 weeks of age (experimental days 98–112, lateC). Both weaning groups comprised of equal number of calves born from primiparous cows (PC) and multiparous cows (MC), with similar pattern of MR and C intake until day 28 of the trial. A step-down weaning approach was followed by gradually reducing MR amount (1.35–0.3 kg/d) over a period of 14 days. In the earlyC group MR amount was reduced from day 28 until day 42. However, the lateC group consumed a constant level of MR (∼ 1300 g DM/d) until day 98 followed by a gradual reduction in MR amount until day 112. All calves received a maximum of 2 kg/day concentrate feed and ad libitum hay over the entire experimental period. The consumption of C started in both weaning groups at around day 21 of the trial. Intake of C increased in earlyC during their weaning period (days 28–42). However, lateC group continued to increase their C intake until day 63 and then consumed a constant level of C (1500–1700 g DM/d) until weaning. When weaning started for lateC group at day 98, C amount was reduced to 1 kg/d to lower the risk of rumen acidification and increase roughage intake. EarlyC group started to consume roughage from day 42, however, the lateC group increased their roughage intake when the MR supply was reduced at day 98. The post-weaning calves’ diet was comprised of hay and a total mixed ration (TMR) containing grass (48%), maize silage (32%), and C (20%). Ingredients and chemical composition of the diets were shown in a companion paper [22].

### Sample collection and preparation

On experimental days 1, 28, 42, 70, 98, 112 and 140 blood and faecal samples were taken from each calf. Blood samples were obtained from *Vena jugularis externa* by needle puncture and collected into tubes (10 ml tubes, Sarstedt, Nürnbrecht, Germany) containing ethylenediaminetetraacetic acid (EDTA). After centrifugation (15 min, 3000 × *g*, Varifuge 3.0, Heraeus, Hanau, Germany), aliquots of plasma samples were stored at − 80 °C until analysis. Faecal samples were taken directly from the calves’ rectum and collected in sample pans. Homogeneous samples were then transferred in sample cups and stored at − 80 °C until the microbiome was analysed. Some of the calves’ samples were discarded due to technical issues as well as during bioinformatic and statistical analysis, thus, resulting in a total of 330 samples over 7 timepoints.

### Faecal bacterial community profiling

The genomic DNA was isolated from the faecal samples (250 mg) using the FastDNA™ SPIN Kit for Feces (MP Biomedical, Solon, OH, USA) according to the manufacturer’s protocol with minor modifications. For an effective lysis of cells, a bead-beating procedure was performed for 40 s at a speed of 6 m/sec using FastPrep®-24 instrument (MP Biomedical), followed by centrifugation at 14,000 *× g* for 15 min. The DNA concentration and quality were accessed using NanoDrop 2000 spectrophotometer (Thermo Fisher Scientific, Waltham, MA, USA).

### Illumina amplicon sequencing and bioinformatic analysis

PCR amplification of the faecal DNA extracts targeting V1-V2 region of bacterial 16 S rRNA gene, and Illumina amplicon sequencing was done as described previously by [79]. Briefly, 20 µl PCR mixture was prepared by adding primers (0.2 µM), dNTP mixture (2.5 mM), PrimeSTAR HS DNA polymerase (2.5 U) and 1 µl DNA template. Forward primers comprised of a linker (2-nt) and a barcode (6-nt) sequence. Additionally, an overhang adapter sequences compatible to the Illumina platform were added to both primers. The PCR conditions comprised of an initial denaturation step for 3 min at 95 °C, followed by 20 cycles involving denaturation for 10 s at 98 °C, annealing for 10 s at 59 °C, extension for 45 s at 72 °C and 72 °C final extension step for 2 min. The resultant PCR product (1 µl) was used in the second PCR step that was performed under similar conditions and comprised of 15 cycles with reverse primer containing additional sequence for integration of Illumina multiplexing sequence and index primers. The PCR products were quality controlled, purified, normalized and sequenced using paired-end (250 bp) Illumina MiSeq sequencing platform.

Bioinformatic analysis of sequencing dataset was performed using QIIME 2 (2019.10) workflow [80]. Briefly, cutadapt (v2.6) was employed within the QIIME 2 for demultiplexing of paired-end (PE) reads according to the barcode sequence of each sample, followed by the trimming of barcodes and primers. The demultiplexed sequences were then quality filtered to remove bases with quality score less than 30, followed by joining of PE reads (mean length 315 ± 14 bp) and removal of non-overlapping regions, chimeras and singletons, thus, resulted in amplicon sequence variant (ASVs) table after DADA2 step. Fourteen faecal samples with < 5000 reads were discarded from the feature table, resulting in a total of 10,221,260 reads for 339 faecal samples with 30,151 ± 1183 reads (mean ± SEM) per sample. The negative control samples had an average of 125 reads per sample and therefore were not included in further analysis. For taxonomic assignments to ASVs, three different reference databases for 16 S rRNA gene were employed i.e., the initial classification was performed using pre-trained naïve Bayesian classifier trained on SILVA 132 clustered at 99% similarity. After initial taxonomic classification, an additional filtration step was employed where the unassigned ASVs and those assigned to chloroplast, cyanobacteria, and non-bacterial taxon were removed, the least abundant features (ASV) with ≤ 0.2% contribution to the total reads per sample were discarded and again the low reads samples (< 5000 reads) were removed, thus, resulting in a total of 8,083,449 reads for 330 faecal samples with 24,495 ± 777 reads (mean ± SEM) per sample and a total of 4,229 unique bacterial ASVs. For taxonomic reassignments of the unique bacterial ASVs, RDP database [81] was used as a reference with naïve Bayesian RDP classifier [82]. The RDP-based taxonomic assignments were then compared with NCBI non-redundant nucleotide database using BLAST [83]. The BLAST results table was filtered with a defined sequence identity threshold for each taxonomic level [84], resulting in removal of taxonomic assignments that fall below the defined threshold; 97.0% (species), 94.5% (genus), 86.5% (family), 82.0% (order), 78.5% (class) and 75.0% (phylum).

For prediction of microbial functional profiles, CowPI was used [85], which is an improved version of PICRUSt, with 16 S rDNA inference for rumen [86]. The functional prediction was based on the16S rRNA gene sequence reads of the differential microbial genera due to the weaning event. Only those level-3 KEGG pathways were used for the downstream analysis that had relative abundance > 1% in at least 50% of the animals within each age group.

### Plasma metabolome analysis

The targeted metabolomic measurements in plasma samples were performed using AbsoluteIDQ p180 kit (Biocrates Life Science AG, Austria) according to the manufacturer’s standard protocol to identify 188 metabolites belonging to 5 compound classes: acylcarnitine, proteinogenic and modified amino acids, glycerophospho- and sphingolipids and hexose. All metabolites were evaluated in absolute concentrations (µmol/l). The assay based on phenylisothiocyanate derivatization in the presence of internal standards followed by FIA-MS/MS (acylcarnitine, hexose, glycerophospho- and sphingolipids) and LC-MS/MS (amino acids, biogenic amines). The experimental measurement technique is described in detail by patent US 8,265,877 B2 [87].

### Statistical analysis

#### Microbiome data

The microbiome dataset standardization was performed with the total sum normalization method, where ASVs read counts were divided by the total number of read in a sample. Alpha-diversity analysis was performed in Calypso v8.84 [88] by rarefying samples to a read depth of 4,702 (lowest read counts). Permutational Analysis of Variance (PERMANOVA) at feature level (ASV) was used to identify the differences between the faecal bacterial community structure between groups. The clustering of samples within/between groups (age, weaning time) was visualized using principal-coordinates analysis (PCO) plots in Primer-e (PRIMER 6.1.16 and PERMANOVA + 1.0.6 [89], that was based on standardized ASV count data and Bray–Curtis as dissimilarity matrix. The significant differences between groups, separated based on PCO analysis, were confirmed using analysis of similarities (ANOSIM) test. Age and weaning-dependent changes in the bacterial diversity and taxonomic composition were tested for statistical significance based on Kruskal–Wallis test in R (https://www.r-project.org; [90]). For multiple comparisons, Dunn’s post-hoc test was used with Benjamini–Hochberg algorithm as *p* value adjustment method and the FDR adjusted *p* < 0.05 was considered significant [91]. The bacterial species-level taxa that were significantly affected by calves age were visualized using heatmap. Heatmap was generated based on hierarchal clustering method using R “gplots” package. The relative abundance table was scaled by row and pairwise distances between species were calculated based on Spearman correlation. These distances were then used to create a dendrogram using average linkage method. Weaning-dependent changes in the predicted metabolic pathways were tested for statistical significance based on Kruskal–Wallis test in R.

#### Metabolome data

Based on targeted metabolomics, a total of 180 metabolic compounds were identified in the plasma samples of calves including free carnitine (1), acylcarnitines (39), amino acids (21), biogenic amines (21), sphingolipids (15), sum of hexoses (1), phosphatidylcholines (76) and lysophosphatidylcholines (14). The latter two metabolite groups were removed from the subsequent analysis as functional aspects of them in calves’ gut are not yet understood. The multivariate and statistical analysis of plasma metabolome data was performed in MetaboAnalyst 5.0 [92]. The data containing the absolute concentrations of 98 compounds was normalized before analysis through log-transformation, mean centering and unit variance scaling method. The maximum separation between groups (age, weaning time, parity of the mother) was explained based on supervised partial least squares-discriminant analysis (PLS-DA). The quality of the PLS-DA models was assessed using Q2 as performance measure and tenfold cross-validation method. Q2 indicates the predictive ability of the model, with high Q2 means good prediction and negative Q2 means overfitting of the model [93]. The dataset containing normalized concentrations of 98 identified metabolic compounds was analysed by one-way ANOVA for age effect and Tukey’s HSD test as post-hoc analysis method. *p* values were adjusted using false discovery rate (FDR) correction and FDR-adjusted *p* < 0.05 was considered statistically significant. To demonstrate the metabolites that were significantly affected by age in calves (VIP > 1, FDR-adjusted *p* < 0.05, ANOVA), a heatmap was generated. For heatmap, the normalized concentration table was scaled by row, pairwise distances between metabolic compounds were calculated based on Euclidean distance measure and ward clustering algorithm. The differential metabolites (DMs) due to the weaning event at each timepoint were selected based on the variable importance in the projection (VIP > 1.0, FDR-adjusted *p* < 0.05 (*t*-test) and earlyC/lateC fold change (FC) > 1.0). The volcano plots with DMs at each timepoint were generated using “ggplot2” package in R. Metabolic pathway analysis (MetPA) was performed based on DMs using *Bos taurus* library as reference [94]. The significantly altered pathways due to the weaning event were selected based on the pathway impact value > 0.1 and FDR-adjusted *p* < 0.01, obtained from pathway enrichment analysis. The associations between bacterial genera, plasma metabolites and morphometric variables of calves were calculated based on Spearman’s rank correlation using cor() function in R and the correlation matrix was visualized using corrplot() function. The correlations with *p* < 0.05 were considered significant.

## Supplementary Information

**Additional file 1. Fig. S1** Age- and weaning-dependent changes in the faecal bacterial compositional profiles of calves. **a** Changes among alpha-diversity index (Shannon index). ^abcde^ Groups that share superscript letters are not significantly different (*p* > 0.05; Dunn’s post-hoc test). Standard deviations are indicated by error bars. **b** Significantly different bacterial phyla. ***Phyla with *p* < 0.001 (age × weaning effect; Kruskal–Wallis test) are shown. **Fig. S2.** Microbial functional predictions using KEGG pathways and the CowPI database. EarlyC/lateC log2(FC) shows differences in level-3 KEGG microbial pathways between d42, d70 and d98 earlyC (blue) and lateC (red) calves. Only metabolic pathways with relative abundance (> 1%) in at least 50% of the animals and FDR adjusted *p* < 0.05 (Kruskal–Wallis test) are shown. **Fig. S3.** Calculation of kynurenine/tryptophan ration at d70 and d98 for early weaned calves (E) and late weaned calves (L). **Fig. S4.** Metabolic pathway analysis based on significantly different plasma metabolites of weaning groups. Circle size indicates pathway impact and colours (yellow to red) show different levels of significance.

**Additional file 2. Table S1.** Average relative abundances of faecal bacterial communities in early- and late-weaned calves. **Table S2.** Average relative concentrations (µmol/L) of plasma metabolites in early- and late-weaned calves. **Table S3.** Spearman’s rank correlations between morphometric variables of calves, differential faecal microbial genera and plasma metabolites of weaning groups.

## Data Availability

Sequences were submitted to European Nucleotide Archive under the accession number PRJEB48866.
