# Supplementary material for: Host metabolome and faecal microbiome shows potential interactions impacted by age and weaning times in calves
Source: Anim Microbiome. 2023 Feb 14;5:12. doi: 10.1186/s42523-023-00233-z (PMC9926800; doi:10.1186/s42523-023-00233-z)
Supplement: Supplementary file 1 — Additional file 1. Fig. S1 Age- and weaning-dependent changes in the faecal bacterial compositional profiles of calves. a Changes among alpha-diversity index (Shannon index). abcde Groups that share superscript letters are not significantly different (p > 0.05; Dunn’s post-hoc test). Standard deviations are indicated by error bars. b Significantly different bacterial phyla. ***Phyla with p < 0.001 (age × weaning effect; Kruskal–Wallis test) are shown. Fig. S2. Microbial functional predictions using KEGG pathways and the CowPI database. EarlyC/lateC log2(FC) shows differences in level-3 KEGG microbial pathways between d42, d70 and d98 earlyC (blue) and lateC (red) calves. Only metabolic pathways with relative abundance (> 1%) in at least 50% of the animals and FDR adjusted p < 0.05 (Kruskal–Wallis test) are shown. Fig. S3. Calculation of kynurenine/tryptophan ration at d70 and d98 for early weaned calves (E) and late weaned calves (L). Fig. S4. Metabolic pathway analysis based on significantly different plasma metabolites of weaning groups. Circle size indicates pathway impact and colours (yellow to red) show different levels of significance. [file 42523_2023_233_MOESM1_ESM.pdf]

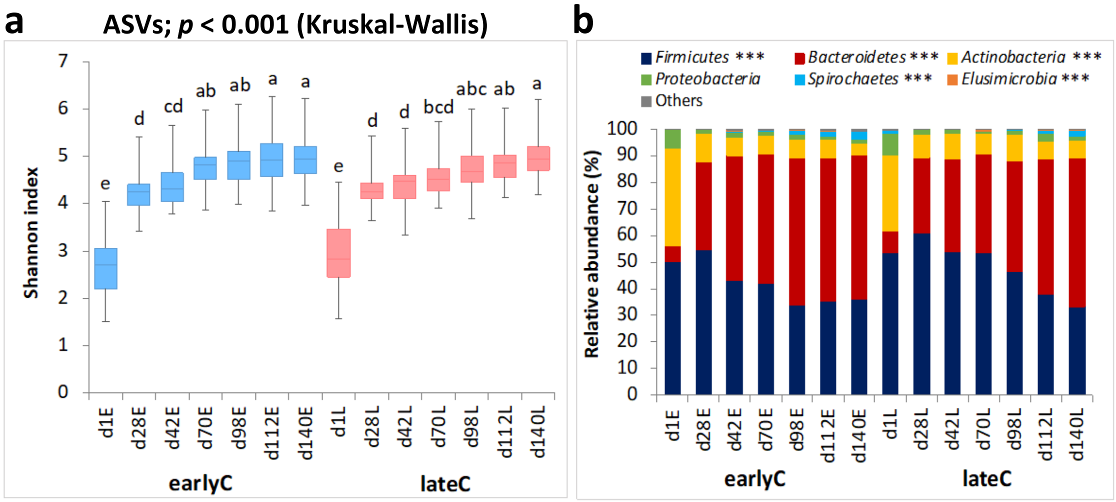

Figure S1 Age- and weaning-dependent changes in the faecal bacterial compositional profiles of calves. (a) Changes among alpha-diversity (Shannon index). abcdeGroups that share superscript letters are not significantly different ($p > 0.05$; Dunn's post-hoc test). Standard deviations are indicated by error bars. (b) Significantly different bacterial phyla. ***Phyla with $p < 0.001$ (age x weaning effect; Kruskal-Wallis test) are shown.

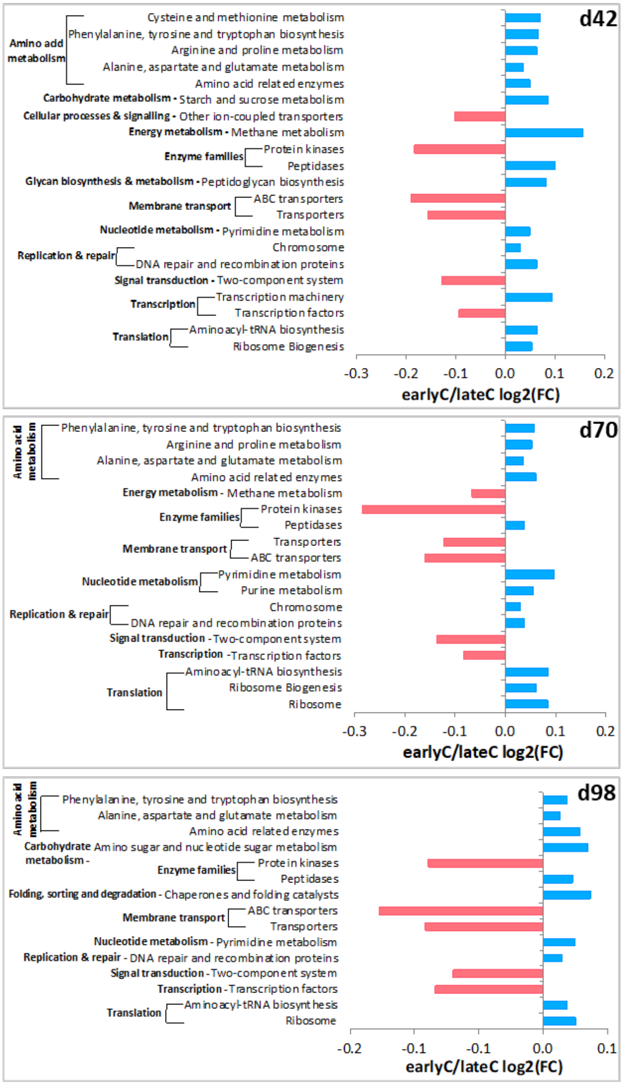

Figure S2. Microbial functional predictions using KEGG pathways and the CowPI database. EarlyC/lateC log2(FC) shows differences in level-3 KEGG microbial pathways between d42, d70 and d98 earlyC (blue) and lateC (red) calves. Only metabolic pathways with relative abundance (> 1%) in at least 50% of the animals and FDR adjusted *p* < 0.05 (Kruskal-Wallis test) are shown.

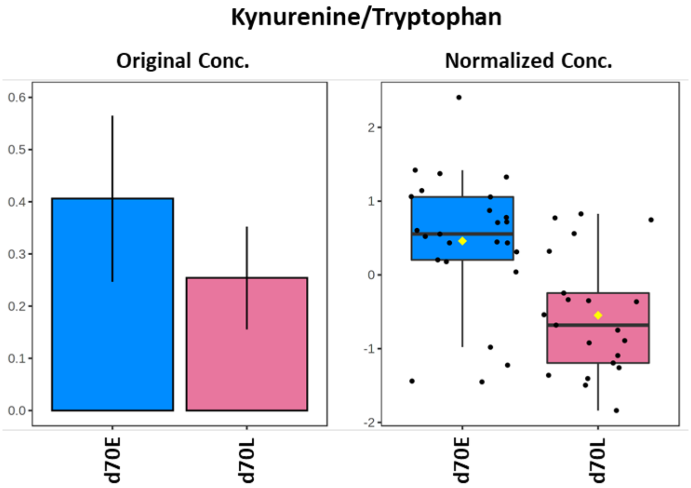

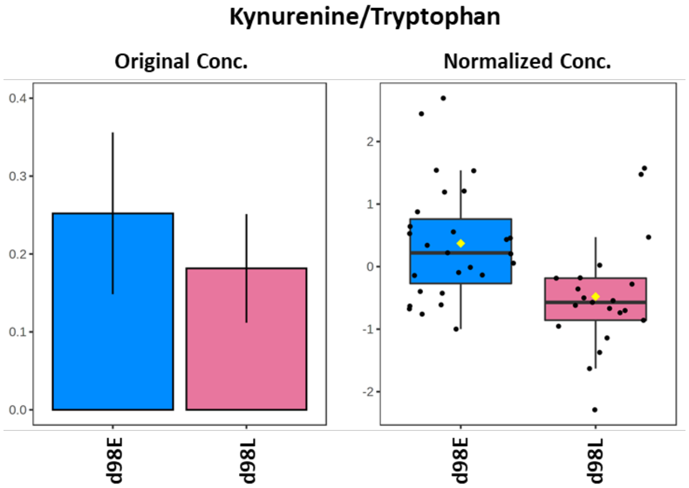

		VIP	FDR (*t*-test)	log2 FC
**d70**	earlyC/lateC	1.5	0.002	0.7
**d98**	earlyC/lateC	1.3	0.010	0.5

Figure S3. Calculation of kynurenine/tryptophan ratio at d70 and d98 for early weaned calves (E) and late weaned calves (L).

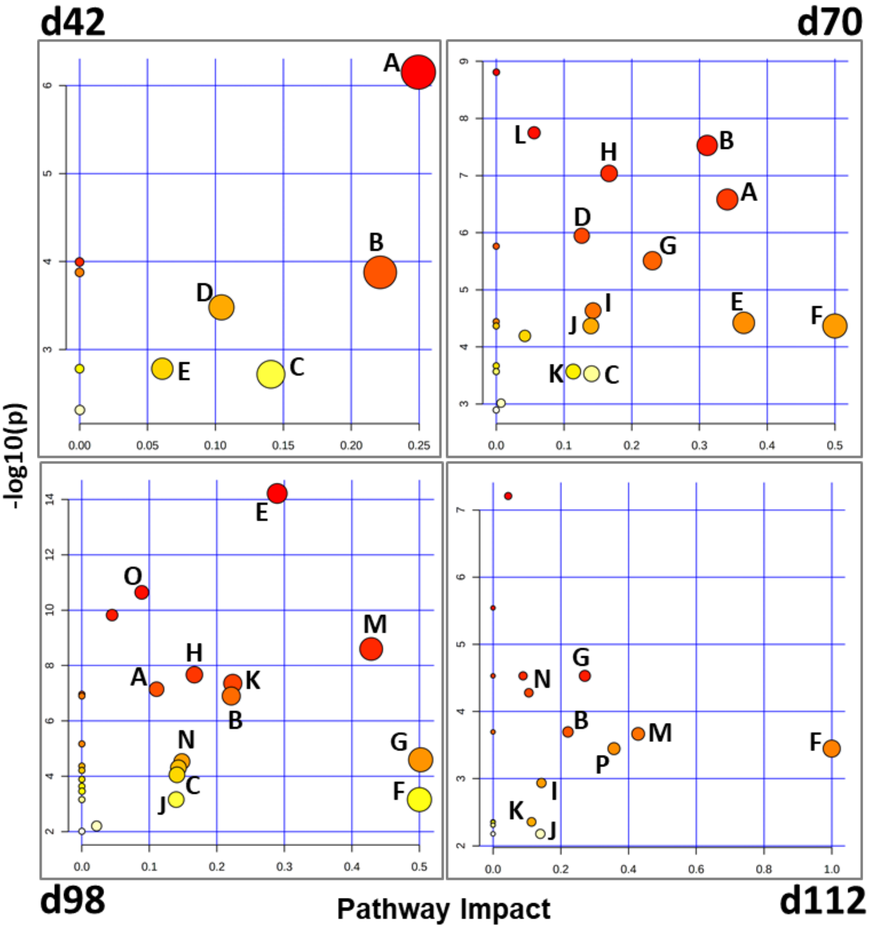

A: Arginine and proline metabolism
B: Histidine metabolism
C: Lysine degradation
D: Cysteine and methionine metabolism
E: Arginine biosynthesis
F: Phenylalanine, tyrosine and tryptophan biosynthesis
G: Glycine, serine and threonine metabolism
H: Aminoacyl-tRNA biosynthesis
I: Tryptophan metabolism
J: Tyrosine metabolism
K: Alanine, aspartate and glutamate metabolism
L: beta-Alanine metabolism
M: Taurine and hypotaurine metabolism
N: Glyoxylate and dicarboxylate metabolism
O: Glutathione metabolism
P: Phenylalanine metabolism

Figure S4. Metabolic pathway analysis based on significantly different plasma metabolites of weaning groups. Circle size indicates pathway impact and colours (yellow to red) show different levels of significance.
